# Fufang Xueshuantong for Diabetic Kidney Disease: A Systematic Review and Meta-Analysis

**DOI:** 10.1155/2020/9326948

**Published:** 2020-11-12

**Authors:** Yu-meng Tan, Jun Hu, Qian Wu, Yi Zhang, Wen-dong Suo, Yu-tong Zhou, He Guo, Qing Ni

**Affiliations:** ^1^Department of Endocrinology, Guang'anmen Hospital of China Academy of Chinese Medical Sciences, Beijing, China; ^2^Department of Cardiovascular, Guang'anmen Hospital of China Academy of Chinese Medical Sciences, Beijing, China; ^3^Beijing University of Chinese Medicine, Beijing, China

## Abstract

**Objective:**

The objective of this meta-analysis was to systematically assess the efficacy and safety of patented Chinese medicine Fufang Xueshuantong (FFXST) for the treatment of diabetic kidney disease (DKD).

**Methods:**

Randomized controlled trials (RCTs) of FFXST for DKD treatment were searched until May 31, 2020, in seven electronic databases: PubMed, Embase, Cochrane Library, CNKI, Wanfang, VIP, and Chinese Biomedical Literature. The Cochrane risk test from the Cochrane Handbook was used as a bias tool to assess the methodological quality, and Review Manager (RevMan) 5.3 was used to analyze the results. Grading of Recommendations Assessment, Development, and Evaluation (GRADE) criteria were used to classify the quality of evidence.

**Results:**

Thirteen RCTs involving 1,186 patients were included. The meta-analysis revealed that the efficacy of FFXST in treatment of DKD was significantly superior to that of the control treatment (*P*=0.0006). The urinary albumin excretion rate (*P* < 0.01), urinary albumin creatinine ratio (*P* < 0.0001), and microalbumin (*P* < 0.0001) were lower in the treatment groups than in the control group. There was also a decrease in low-density lipoprotein cholesterol (*P* < 0.0001), serum triglyceride (*P*=0.001), and C-reactive protein (*P* < 0.0001) in the treatment groups compared with those in the control group. No significant difference in hemoglobin A1c level (*P*=0.76) and systolic blood pressure (*P*=0.34) was noted between the treatment and control groups. Three studies reported adverse events, including dizziness and intolerance. In the other 10 trials, adverse events were not mentioned.

**Conclusion:**

FFXST appears to be effective in the treatment of DKD. However, the low methodological quality of the RCTs suggests that larger, better-designed RCTs are required to verify the clinical eﬀectiveness and safety of FFXST.

## 1. Introduction

Diabetic kidney disease (DKD) is one of the most important microvascular complications of diabetes, as well as a key cause of end-stage renal disease (ESRD). It also increases the risk of cardiovascular disease and all-cause death in patients with diabetes [1, 2]. With the incidence of diabetes increasing annually, the number of DKD cases is also increasing. Approximately 20%–40% of patients with diabetes also have DKD [3]. The risk factors of DKD include age, disease course, blood pressure, obesity (especially abdominal obesity), blood lipid, uric acid, and environmental pollutants [4]. The main clinical manifestations of DKD are proteinuria and (or) impaired glomerular filtration rate (GFR) [3, 5].

Because the occurrence and development of DKD is the result of multifactor interactions, the treatment involves targeting hypoglycemia and hypotension as well as the reduction of proteinuria. Previously, renin-angiotensin-aldosterone system (RASS) inhibitors (angiotensin-converting enzyme inhibitors [ACEI] and angiotensin II receptor blockers [ARB] drugs) have had the most clinical evidence and are recommended as first-line drugs for the treatment of DKD; however, the renal protection effect of RASS inhibitors is limited. More recently, sodium-glucose cotransporter 2 inhibitors have also been recommended for the treatment of DKD. Despite this, a large number of patients with DKD progress to ESRD or die from complications of vital organs outside the kidney annually; thus, it is necessary to develop additional treatment methods to counter DKD [6].

In traditional Chinese medicine, DKD belongs to the categories “cloudy urine” and “edema” [7]. In recent years, many Chinese herbal extracts and Chinese patent medicines have demonstrated the reduction of proteinuria and the improvement of renal function in the treatment of DKD. Among them, the Chinese patent medicine Fufang Xueshuantong (FFXST) has been widely used in the treatment of DKD and other diabetic microvascular complications in China. FFXST is composed of *Notoginseng Radix*, *Radix Astragali*, *Salvia Miltiorrhiza,* and *Scrophularia Ningpoensis*. Studies [8–10] have shown that FFXST has a protective effect on the kidneys of diabetic rats, can reduce oxidative stress injury, regulate the RASS system, promote podocyte repair, and improve microcirculation and antiplatelet aggregation.

Although several clinical trials have suggested the efficacy of FFXST for DKD, most of the trials have been single-center, including small cohorts and highly different treatment schemes. Hence, it is difficult to verify the clinical efficacy of these treatment strategies. Therefore, the goal of this meta-analysis was to assess the efficacy and safety of FFXST for the treatment of DKD, providing evidence for clinical practice.

## 2. Materials and Methods

### 2.1. Search Strategy

We searched seven electronic databases, including PubMed database, Embase database, Cochrane Library, China National Knowledge Infrastructure database, Wanfang Database, Chinese Science Journal Database, and the Chinese Biomedical Database. We retrieved studies from all of these databases published before May 31, 2020. Our search keywords were as follows: “diabetic kidney disease” OR “diabetic nephropathy” AND “fufang xueshuantong” OR “compound xueshuantong” AND “randomized controlled trial,” “controlled clinical trial,” “random,” “randomly,” “randomized” OR “control.” Furthermore, we manually searched additional relevant publications according to reference lists from the resulting publications. Different search strategies were applied to Chinese and foreign language databases, without restriction on language or publications.

### 2.2. Inclusion Criteria

1. Types of trials: randomized controlled trials (RCTs) using FFXST monotherapy or combination therapy with western medicine for the treatment of DKD were included.
2. Types of patients: regardless of the type of diabetes mellitus (DM), stage of the DKD (Mogensen staging criteria), age, gender, or race, we recruited patients who were diagnosed with DKD by clearly defined or internationally recognized criteria.
3. Types of interventions: the experimental group was treated with FFXST monotherapy irrespective of the dosage form or combined with conventional western medicine (ACEI/ARB). There was no limit to interventions in control groups, whether placebo or ACEI/ARB. Additionally, both groups received routine treatment, such as treatment to lower blood pressure, controlling blood glucose, and regulating serum lipids.
4. Types of outcomes: all included studies that reported at least one of the following outcomes: total effective rate or proteinuria indicators.

### 2.3. Exclusion Criteria

1. Interventions that included other traditional Chinese medicine (TCM) therapies, such as Chinese patent medicine, TCM decoction, herbal extracts, or acupuncture were excluded
2. Studies with erroneous or incomplete data were excluded
3. Duplicate publications were excluded

### 2.4. Data Extraction

Two researchers extracted the information independently. The data included study ID, baseline patients, disease data, interventions, and outcomes (e.g., sample size, age, gender, type of DM, stage of DKD, interventional measures, treatment duration, reporting of adverse events, and outcome measures). Discrepancies were resolved by discussion with other authors.

### 2.5. Quality Assessment

Two researchers assessed the risk of bias in trials based on the Cochrane Handbook for the methodological quality of the included studies. We applied the RevMan5.3 to assess the following six items: random sequence generation (selection bias), allocation concealment (selection bias), blinding of participants and personnel (performance bias), blinding of outcome assessment (detection bias), incomplete outcome data (attrition bias), selective reporting (reporting bias), and other sources of bias such as baseline comparability of subjects and sample size. We also used the Grading of Recommendations, Assessment, Development, and Evaluations (GRADE) approach to assess the quality of evidence for each outcome by GRADEpro GDT software. This classifies evidence as high, moderate, low, or very low quality. Discrepancies were resolved by a third party (Qing Ni).

### 2.6. Data Analysis

RevMan 5.3 software (Cochrane Collaboration, Oxford, UK) was applied for statistical analysis. Dichotomous data were presented as relative risk (RR), and continuous data were included as the mean difference (MD) or standardized mean difference (SMD) and both included a 95% confidence interval (CI). The heterogeneity evaluations were conducted using a Chi^2^ test. The fixed-effects model was used when the heterogeneity was significant (*P* > 0.10, I2 ≤ 50%); otherwise, a randomized effects model was used (i.e., when *P* < 0.10, I2 ≥ 50%). The possible sources of heterogeneity were explored by sensitivity analysis and subgroup analysis. Publication bias was tested using funnel plots when the number of experiments was ≥10 [11].

### 2.7. Outcomes

The primary outcome indicator was a total effective rate, which was based on changes in symptoms and the level of proteinuria [12]. The total effective rate was categorized as significantly effective cases (urinary albumin excretion rate [UAER] returned to normal levels or decreased by more than 50%, with an obvious improvement in symptoms), effective cases (UAER decreased by less than 50%, improvement in symptoms), or ineffective cases (no improvement in either UAER and symptoms).

The secondary outcomes included the proteinuria indicators UAER, urinary albumin creatinine ratio (ACR), and microalbumin (mAlb); the renal function indicators estimated glomerular filtration rate (eGFR), blood urea nitrogen (BUN), and serum creatinine (Scr); hemoglobin A1c (HbA1c); low-density lipoprotein cholesterol (LDL-C); triglyceride (TG) level; blood pressure indicators; inflammatory indicators.

## 3. Results

### 3.1. Study Search and Selection

**Figure 1:**
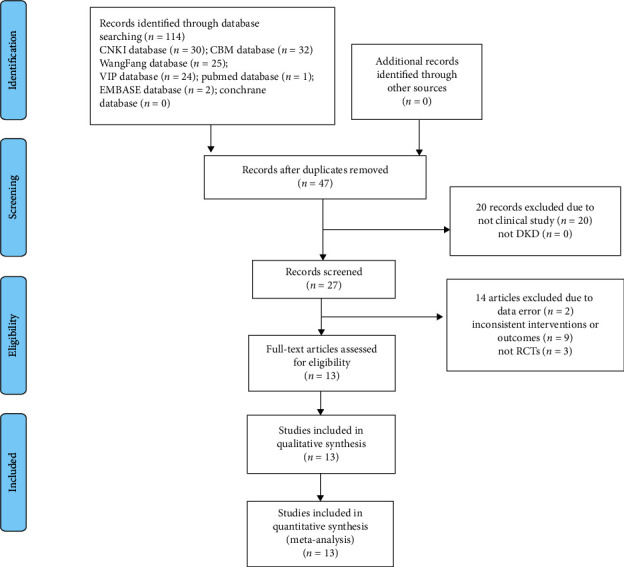
Flowchart of the literature retrieval.

Initially, a total of 114 publications were identified from the seven electronic databases. After removing 67 duplicate publications, we excluded 20 nonclinical studies by reading the titles and abstracts. After a full-text review, we excluded two studies with significant data errors, three nonrandomized controlled studies, and nine interventions or outcome indicators that did not meet inclusion criteria. Finally, 13 studies were included in this meta-analysis. The retrieval process is shown in Figure 1.

### 3.2. Characteristics of the Included Studies

Characteristics of the 13 studies [13–25] are summarized in Table 1. All the included studies were published between 2009 and 2019. The 13 RCTs involved 1186 subjects (592 in treatment groups and 594 in control groups), and the sample size for each study ranged from 48 to 130 subjects. In terms of the disease type and stage, patients with type 2 diabetes (T2DM) were included in eight studies, whereas the remaining five studies did not report in detail the type of diabetes patients included. Except for one study [14] that did not report the DKD stage, the remaining 12 studies included subjects who were DKD patients in Mogensen III according to the Mogensen stage. The subject's DKD diagnosis was clear in 13 studies. All studies included patients who met the diagnostic criteria for DKD, nine of which used the criteria of the World Health Organization (WHO) DM diagnostic criteria [26] and Mogensen diagnostic [27]. One study [18] referred to the American Diabetes Association criteria combined with the Epidemiology and Diagnostic Criteria of Diabetic Nephropathy [28]. Another [16] used the WHO DM1999 and pathological diagnosis of renal biopsy. The diagnosis of DKD in one study [14] was based on internal diagnostic criteria for diabetes combined with symptoms of proteinuria and history of diabetic retinopathy and the internal DKD diagnostic criteria were used in another study [24].

Compared with the control group, treatment groups in six RCTs [16, 17, 22–25] were treated with FFXST monotherapy, whereas treatment groups in the other seven RCTs received FFXST combined with ACEI/ARB. All patients in both groups were treated with conventional hypoglycemic therapy. The duration of the trials ranged from 8 weeks to 24 weeks.

Only two studies used the total effective rate based on changes in symptoms and urinary protein levels as the main outcome indicators. In terms of proteinuria indicators, five studies reported UAER, six studies reported ACR, and six studies reported mAlb. Additionally, we also used BUN, HbA1c, standard bicarbonate (SBP), TG, LDL-C, and C-reactive protein (CRP) as secondary outcome indicators. Adverse events were not mentioned in three studies [15, 17, 21], and none of the studies reported a decrease in the quality of life or took adverse indicators (e.g., deterioration rate, access to dialysis rate, etc.) as outcome measures.

### 3.3. Risk of Bias in the Included Studies

**Figure 2:**
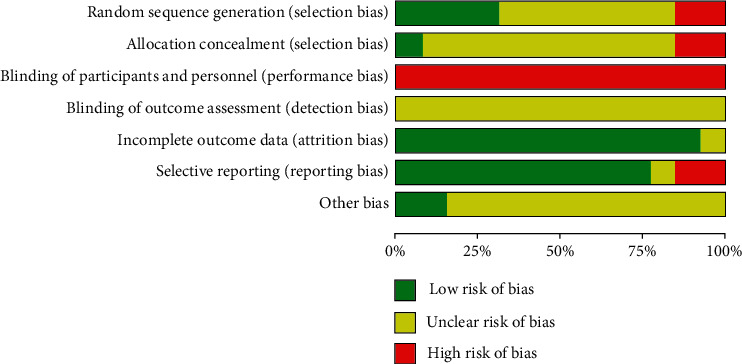
Risk of bias graph.

**Figure 3:**
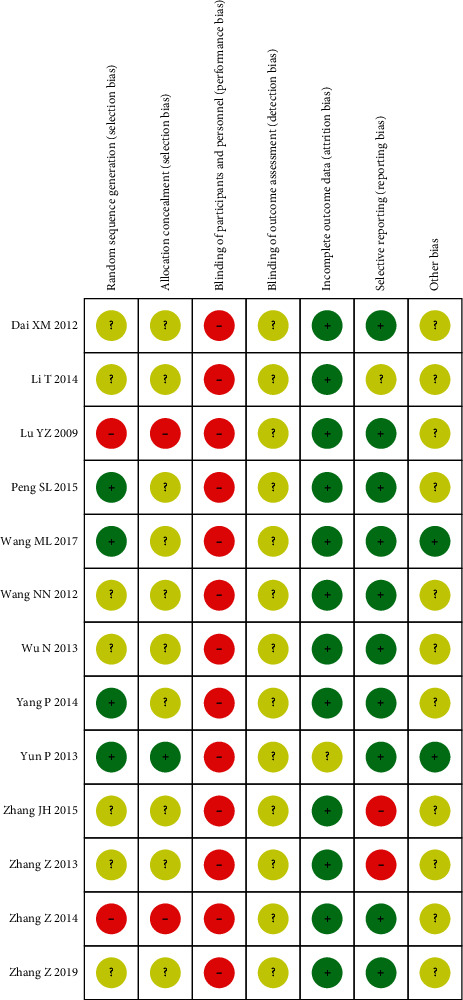
Risk of bias summary.

The methodological quality assessment of the 13 RCTs is shown in Figures 2 and 3. Two studies [16, 17] adopted methods of randomization using a random number table; one study [20] used mechanical random sampling, and seven studies [13, 14, 18, 19, 22, 23, 25] only mentioned “randomization” but did not describe specific methods. The remaining three RCTs had a high risk of selection bias, because two [15, 21] followed the order of medical treatment, and one [24] followed the case number. None of the 13 studies were double-blind, and no study indicated details on allocation concealment or sample size calculations. Two studies [22, 23] showed high-risk bias in selective reporting. Baseline information was similar for different groups of subjects in all 13 studies. In short, the quality of all RCTs was generally low and contained a risk of bias.

### 3.4. Effects of the Interventions

#### 3.4.1. Total Effective Rate

**Figure 4:**
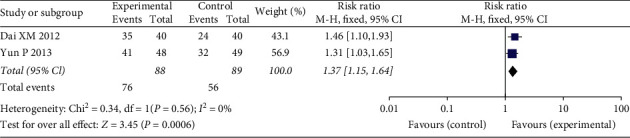
Forest plots of FFXST effects on total effective rate.

The total effective rate was reported in two studies, and the results indicated significant differences between the two groups. These trials exhibited nonsignificant heterogeneity (*χ*^2^ = 0.34, *P*=0.56, I2 = 0%); thus, the fixed-effects model was used for statistical analysis. The total effective rate of the treatment groups was superior to that of the control groups (*N* = 177, RR = 1.37, 95% CI: 1.15–1.64, *Z* = 3.45, *P*=0.0006) (Figure 4).

#### 3.4.2. UAER

**Figure 5:**
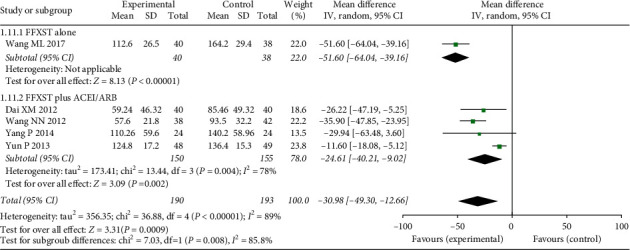
Forest plots of FFXST effects on UAER.

Five studies (Dai XM, 2012,Wang ML, 2017, Wang NN, 2012, Yang P, 2014, and Yun P, 2013) evaluated changes in UAER (Figure 5) according to indictors involving 383 patients (MD = −30.98, 95% CI: −49.30–12.66, *Z* = 3.31, *P* = 0.0009). There was significant heterogeneity among the studies (*χ*^2^ = 36.88, *P* < 0.00001, I2 = 89%), and a random effects model was used for combined analysis.

To further compare the differences in UAER between FFXST combined with conventional western medicine and control groups, subgroup analysis was performed. In one study [17], FFXST monotherapy in the treatment group was compared with that of the control group (MD = −51.60, 95% CI: −64.04–39.16, *Z* = 8.13, *P* < 0.00001). FFXST combined with ACEI/ARB was compared with control groups in the remaining four studies (*N* = 305, MD = −24.61, 95% CI: −40.21–9.02, *Z* = 3.09, *P*=0.002), without significant heterogeneity (*χ*2 = 13.44, *P*=0.004, I2 = 78%). Sensitivity analysis suggested that the heterogeneity between the subgroups decreased significantly after removal of one study [21] (*χ*2 = 0.66, *P*=0.72, I2 = 0%). The test method and kit used in this study for the detection of UAER indicators may have been a source of heterogeneity.

#### 3.4.3. ACR

**Figure 6:**
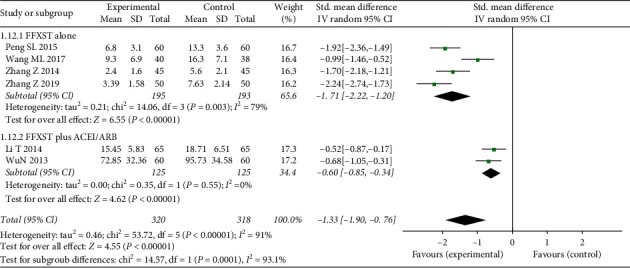
Forest plots of FFXST effects on ACR.

Six studies involving a total of 560 patients reported ACR as an outcome (SMD = −1.33, 95% CI: −1.90–0.76, *Z* = 4.55, *P* < 0.0001) (Figure 6). A random effects model was used because of the significant heterogeneity among studies (*χ*^2^ = 53.72, *P* < 0.00001, I2 = 91%).

Among these, two studies compared the ACR outcomes of FFXST plus ACEI/ARB with those of the control group (*N* = 250, SMD = −0.6, 95% CI: −0.85–0.34, *Z* = 4.62, *P* < 0.00001) without heterogeneity (*χ*^2^ = 0.35, *P*=0.55, I2 = 0%). Four studies compared ACR outcomes between FFXST alone and control groups (*N* = 310, SMD = −1.71, 95% CI: −2.22–−1.20, *Z* = 6.55, *P* < 0.00001), with heterogeneity (*χ*^2^ = 14.06, *P*=0.003, I2 = 79%). Further sensitivity analysis showed that, after the exclusion of one study [17], the subgroup sensitivity decreased (*χ*^2^ = 14.06, *P*=0.032, I2 = 12%). The sources of heterogeneity may have been related to the conventional treatment regimen adopted in the study, where ACEI/ARB drugs were preferred for patients with hypertension, leading to some patients belonging to the FFXST plus ACEI/ARB subgroup, rather than FFXST subgroup alone.

#### 3.4.4. mAlb

**Figure 7:**
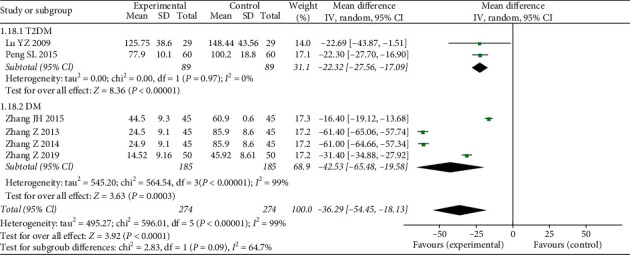
Forest plots of FFXST effects on mAlb.

Six studies reported mAlb as an outcome (*N* = 548, MD = −36.29, 95% CI: −54.45–18.13, *Z* = 3.92, *P* < 0.0001) (Figure 7) with heterogeneity (*χ*^2^ = 596.01, *P* < 0.00001, I2 = 99%) and the use of a random effects model. Then, we performed a subgroup analysis to compare the effects of different diabetes types on the results. The subjects in two studies were T2DM patients, and there was no heterogeneity between these groups (*χ*^2^ = 0.00 *P*=0.97, I2 = 0%). The results showed that the mAlb changes were statistically different between treatment and control groups (*N* = 178, MD = -22.32, 95% CI: −27.56–17.09, *Z* = 8.36, *P* < 0.00001). The other four studies did not limit the type of diabetes and had significant heterogeneity (*χ*^2^ = 564.54 *P* < 0.00001, I2 = 99%). The changes in mAlb were statistically different between the two groups (*N* = 270, MD = −42.53, 95% CI: −65.48.56–19.58, *Z* = 3.63, *P*=0.0003). We speculated that the source of heterogeneity might have been the different test method of mAlb, such as radioimmunoassay or enzyme-linked immunosorbent assay.

#### 3.4.5. BUN

**Figure 8:**
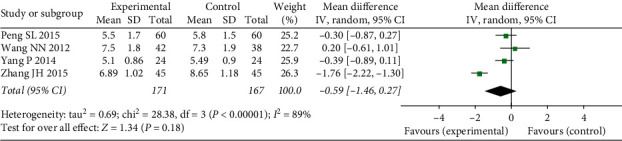
Forest plots of FFXST effects on BUN.

Four studies evaluated the change in BUN (Peng SL, 2015, Wang NN, 2012, Yang P, 2014, and Zhang JH, 2015) and exhibited heterogeneity (*χ*^2^ = 28.38, *P* < 0.00001, I2 = 89%); thus, a random effects model was used. There was no significant difference between treatment and control groups (*N* = 338, MD = −0.59, 95% CI: −1.46–0.27, *Z* = 1.34, *P*=0.18) (Figure 8). Sensitivity analysis showed that heterogeneity decreased after the removal of one study (Zhang JH, 2015) (*χ*^2^ = 1.51, *P*=0.47, I2 = 0%). Considering that the study inclusion criteria did not limit the diabetes type and the subjects of the other three studies were all T2DM patients, diabetes type may have been a source of heterogeneity.

#### 3.4.6. Glycemic Control

**Figure 9:**
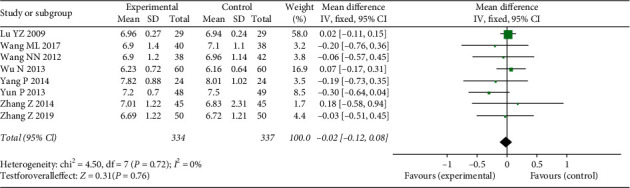
Forest plots of FFXST effects on HbA1c.

In this review, we mainly assessed HbA1c. This indicator was reported in eight studies, involving 671 people. A fixed-effect model was used because there was no heterogeneity between studies (*χ*^2^ = 4.5, *P*=0.72, I2 = 0%). The results indicated that there was no significant difference in HbA1c between two groups in the meta-analysis (MD = −0.02, 95% CI: −0.12–0.08, *Z* = 0.31, *P*=0.76) (Figure 9).

#### 3.4.7. Blood Lipid

**Figure 10:**
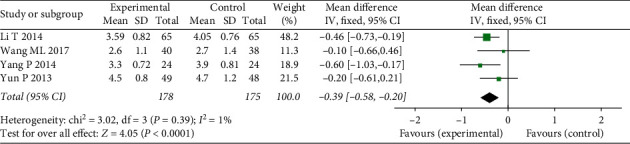
Forest plots of FFXST effects on LDL-C.

This meta-analysis evaluated two blood lipid indicators, LDL-C and TG. Four studies reported that the LDL-C indictor had low heterogeneity (*χ*^2^ = 3.02, *P*=0.39, I2 = 1%), and a fixed-effect model was adopted. It was determined that the LDL-C level of treatment groups was lower than that of the control groups (MD = −0.39, 95% CI: −0.58–0.20, *Z* = 4.05, *P* < 0.0001) (Figure 10).

**Figure 11:**
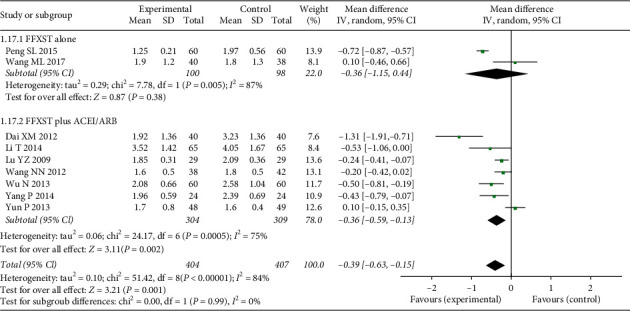
Forest plots of FFXST effects on TG.

Nine studies reported the TG indictor and lacked heterogeneity (*χ*^2^ = 51.42, *P* < 0.00001, I2 = 84%). Therefore, a fixed-effects model was used for data analysis. The results revealed that the TG level in treatment groups was lower than that in control groups (*N* = 811, MD = −0.39, 95% CI: −0.63–0.15, *Z* = 3.21, *P*=0.001) (Figure 11). To compare the differences in TG between FFXST plus western medicine and control groups, subgroup analysis was performed. Two of the studies compared changes in TG in FFXST monotherapy treatment with that of control groups, and the results revealed there was no statistical difference TG improvement between the two groups (*N* = 198, MD = −0.36, 95% CI: −1.15–0.44, *Z* = 0.87, *P*=0.38) with heterogeneity (*χ*^2^ = 7.78, *P*=0.005, I2 = 87%). The difference between the two studies may have been caused by the difference in intervention measures in the control groups. The control group in one study (Peng S. L., 2015) used valsartan 80 mg QD (daily), whereas the control group in the other study (Wang M. L., 2017) used a blank control.

The difference in TG between the FFXST plus ACEI/ARB and control groups was compared in the other seven studies. The results indicated there was no statistical difference in TG improvement between the two groups (*N* = 613, MD = −0.36, 95% CI: −0.59–0.13, *Z* = 3.11, *P*=0.002) with heterogeneity (*χ*^2^ = 24.17, *P*=0.0005, I2 = 75%). Sensitivity analysis showed that heterogeneity decreased after the removal of two studies (Dai XM, 2012, and Yun P, 2013) (*χ*^2^ = 4.05, *P*=0.40, I2 = 1%). However, the TCM syndrome in the subjects in the two studies was the syndromes of Qi and Yin deficiency combined with stasis, and the remaining studies were not limited to TCM syndromes. Thus, TCM syndromes may have been the source of heterogeneity.

#### 3.4.8. Blood Pressure

**Figure 12:**
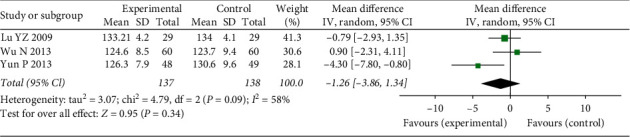
Forest plots of FFXST effects on SBP.

We mainly used the SBP indictor to evaluate BP changes. This indicator was reported in three studies (*N* = 275, MD = −1.26, 95% CI: −3.86–1.34, *Z* = 0.95, *P*=0.34) (Figure 12) with heterogeneity (*χ*^2^ = 4.79, *P*=0.09, I2 = 58%). Sensitivity analysis showed that after one study (Yun P, 2013) was removed, there was no heterogeneity (*χ*2 = 0.74, *P*=0.39, I2 = 0%). The source of heterogeneity may have been the difference in TCM syndromes.

#### 3.4.9. Inflammatory Index

**Figure 13:**
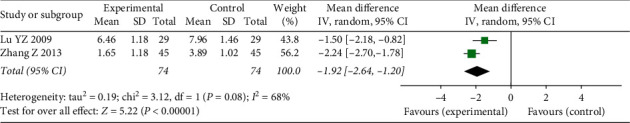
Forest plots of FFXST effects on CRP.

Three studies assessed the inflammatory indexes, including CRP, HS-CRP, IL-6, and TNF-*α*. This review mainly evaluated CRP. Two studies reported the CRP indictor, with heterogeneity (*χ*^2^ = 3.12, *P*=0.08, I2 = 68%). The improvement in CRP was significantly different between the two groups (*N* = 148, MD = −1.92, 95% CI: −2.64–1.20, *Z* = 5.22, *P* < 0.00001) (Figure 13).

#### 3.4.10. Adverse Events

Three of the 13 RCTs mentioned adverse events in treatment groups. No adverse reactions occurred in the treatment group one study (Lu YZ, 2009). In the treatment group of two studies (Wang ML, 2017, and Yun P, 2013), five patients had dizziness and three patients had a poor appetite. Adverse events were not reported in the other 10 RCTs; therefore, the safety of the FFXST therapy needs further evaluation.

#### 3.4.11. Publication Bias

We could not conduct the funnel plot analysis for the detection of publication bias because of an insuﬃcient number of experiments.

### 3.5. Grade Assessment

According to the GRADE, the quality of evidence was rated as moderate for the primary outcome, and the secondary outcomes were rated as low, very low, or moderate (Table 2).

## 4. Discussion

DKD is one of the common microvascular complications of DM. Early prevention and treatment can delay the occurrence and progression of DKD, which is of great significance to the improvement, survival rate, and quality of life of diabetic patients [29]. Clinical practice shows that TCM has the characteristics of multitarget, multipathway, and low adverse reaction [30–32] and has a great potential in the intervention of DKD. FFXST is a Chinese patent medicine approved by the State Food and Drug Administration of China and has been widely used for the treatment of DKD. This meta-analysis suggests that FFXST may be a safe and effective treatment for DKD. This meta-analysis revealed that FFXST for DKD was superior to the treatments provided the control group in total effective rate. Additionally, FFXST exhibited advantages in improving proteinuria indicators (UAER, ACR, and mAlb), blood lipid (LDL-C, TG), and inflammatory index (CRP), but not in lowering BUN or HbA1c levels or blood pressure in DKD patients. However, it has been reported that FFXST has a certain antihypertensive effect, which needs to be confirmed by further studies in the future [33].

There were many limitations to our meta-analysis. Although the dosage and form of FFXST used in the 13 studies were consistent, the treatment periods, DM type, and DKD stage of patients were not similar among the RCTs. Furthermore, the methodological quality of the studies was generally low, and the sample size was not reported in the 13 RCTs. All of the previously mentioned factors might have negatively affected the reliability of the research results.

Regarding the outcome, only two in the 13 RCTs reported the total effective rate, which was the primary outcome in our review. The improvement of the symptoms of patients is an important part of the evaluation of the efficacy of DKD treatment, but only one study (Lu YZ, 2009) reported the change of symptom score of patients before and after treatment. Thus, we could not evaluate the improvement effect of FFXST on the symptoms of patients. It was necessary to standardize the DKD efficacy evaluation system in clinical trials, which could have improved the reliability of the analysis. One study (Dai XM, 2012) also showed that FFXST could improve hemorheology. However, such reports were rare, and more pharmacological and clinical studies are needed to verify the mechanism of FFXST in the treatment of DKD. Clinical events are often recommended as primary outcome indicators for clinical studies; however, no trials assessed the incidence of DKD clinical endpoint events (death/entry to ESRD) or other adverse indicators in our study, which may not be conducive to explain the effect of FFXST for DKD. The GRADE results showed that the evidence quality of the total effective rate and LDL-C level was moderate, and the quality of the remaining outcomes was low or very low. This is mainly due to the fact that most of the included studies did not use blind methods or the large heterogeneity between studies. Therefore, more rigorous clinical studies are still needed to confirm the efficacy of FFXST in the treatment of DKD.

Follow-up and adverse event reports were insufficient among the 13 studies, with only three studies reporting adverse events and one study reporting a follow-up; thus, this meta-analysis was unable to assess the long-term efficacy and safety of FFXST for DKD.

## 5. Conclusions

Our meta-analysis suggested that the Chinese patent medicine FFXST was superior to that of the treatment of the control group in the improvement of total effective rate, reduction of proteinuria, and lowered blood lipid. DKD patients, especially who are in the stage of Mogensen III, accompanied by abnormalities in indicators of UAER, ACR, mAlb, LDL-C, TG, and CRP, can be treated with FFXST or combined with western medicine. However, FFXST may not be an optimizing option to improve abnormal indicators of SBP, HbA1c, and BUN in DKD patients.

However, the long-term efficacy and safety of FFXST for DKD is uncertain because most studies included in this review were of low quality, having small sample sizes and high heterogeneity. Thus, high-quality, large-scale, and multicenter RCTs are needed to validate the current results.

## Figures and Tables

**Table 1:** Characteristics of the included studies.

No.	Study ID	Sample size (T/C)	Average age (T)	Average age (C)	Sex (M/F)	Type of DM	DM duration (year)	Stage of DKD	Diagnostic criteria	Intervention (T)	Intervention (C)	Treatment duration (weeks）	Adverse event report	Outcomes
1	Dai XM [13]	40/40	51.7 ± 5.2	52.2 ± 1.3	T: 25/15 C: 23/17	No restriction	T: 12.4 ± 6.1 C: 13.1 ± 5.9	Mogensen III	WHO DM1999 +MDC	FFXST (1.5 g, tid) +ACEI (Benazepril 10 mg, qd)	ACEI (Benazepril 10 mg, qd)	12 w	NO	Total effective rate, UAER, TG
2	Li T [14]	65/65	46.5 ± 1.38	48.2 ± 1.0	T: 41/24 C: 37/28	T2DM	T:≥5 C:≥5	Not report	Internal diagnostic criteria for diabetes combined with symptoms of proteinuria and history of diabetic retinopathy	FFXST (1.5 g, tid) +ACEI/ARB	ACEI/ARB	12 w	NO	ACR, TG, LDL-C
3	Lu YZ [15]	29/29	48.73 ± 4.26	50.37 ± 4.8	T: 15/15 C: 14/16	T2DM	T: 5.78 ± 1.05 C: 5.85 ± 0.98	Mogensen III	WHO DM1999 + MDC	FFXST (1.5 g, tid) +ACEI (Benazepril 10–20 mg, qd)	ACEI (Benazepril 10–20 mg, qd)	12 w	YES	mAlb, HbA1c, TG, SBP, CRP
4	Peng SL 2015 [16]	60/60	59.89 ± 8.24	58.28 ± 8.5	T: 34/28 C: 26/26	T2DM	T: 7.2 ± 3.1 C: 7.5 ± 2.9	Mogensen III	WHO DM1999+pathological diagnosis of renal biopsy	FFXST (1.5 g, tid)	ARB (Valsartan 80 mg, qd)	8 w	NO	ACR, mAlb, BUN, TG
5	Wang ML 2017 [17]	40/38	54.5 ± 6.6	56.1 ± 7.1	T: 22/18 C: 19/19	T2DM	**—**	Mogensen III	WHO DM1999 + MDC	FFXST (1.5 g, tid)	Blank	12 w	YES	UAER, ACR, HbA1c, TG, LDL-C
6	Wang ML [18]	38/42	55.4	54.5	T: 17/21 C: 18/24	T2DM	T: 5–17 months C: 4–17 months	Mogensen III	ADA2009+ EDCDN	FFXST (1.5 g, tid) +ARB (Losartan potassium 50 mg, qd)	ARB (Losartan potassium 50 mg, qd)	24 w	NO	UAER, BUN, HbA1c, TG
7	Wu NN 2012 [19]	60/60	56.3 ± 11.5	58.7 ± 10.2	T: 35/25 C: 34/26	T2DM	**—**	Mogensen III	WHO DM1999 + MDC	FFXST (1.5 g, tid) +ARB (Valsartan 80 mg, qd)	ARB (Valsartan 80 mg, qd)	12 w	NO	ACR, HbA1c, TG, SBP
8	Yang P 2014 [20]	24/24	52.1 ± 5.3	53.6 ± 4.9	T: 13/11 C: 12/12	T2DM	**—**	Mogensen III	WHO DM1999 + MDC	FFXST (1.5 g, tid) +ACEI (Benazepril 10 mg, qd)	ACEI (Benazepril 10 mg, qd)	12 w	NA	UAER, BUN, HbA1c, TG, LEL-C
9	Yun P 2013 [21]	51/51	53.5 ± 6.4	55.1 ± 7.2	T: 32/19 C: 29/22	T2DM	**—**	Mogensen III	WHO DM1999 + MDC	FFXST (1.5 g, tid) +ARB (Losartan 50 mg, qd)	ARB (Losartan 50 mg, qd)	12 w	YES	Total effective rate, UAER, HbA1c, TG, LEL-C, SBP
10	Zhang JH [22]	45/45	**—**	**—**	50/40	No restriction	8.5	Mogensen III	WHO DM1999 + MDC	FFXST (1.5 g, tid)	Blank	12 w	NO	mAlb, BUN
11	Zhang Z [23]	45/45	**—**	**—**	50/40	No restriction	8.5	Mogensen III	WHO DM1999 + MDC	FFXST (1.5 g, tid)	Blank	12 w	NO	mAlb, CRP
12	Zhang Z [24]	45/45	48.9 ± 10.1	49.4 ± 9.6	T: 31/14 C: 29/16	No restriction	**—**	Mogensen III	The internal DKD diagnostic criteria	FFXST (1.5 g, tid)	Blank	12 w	NO	ACR, mAlb, HbA1c
13	Zhang Z [25]	50/50	58.9 ± 10.18	59.2 ± 9.7	60/40	No restriction	**—**	Mogensen III	The internal DKD diagnostic criteria	FFXST (1.5 g, tid)	Blank	12 w	NO	ACR, mAlb, HbA1c

*T*: treatment group; *C*: control group; M/F: men/female; DM: diabetes mellitus; DKD: diabetic kidney disease; WHO DM1999: World Health Organization diabetes mellitus diagnostic criteria (1999); MDC: Mogensen diagnostic criteria; ADA2009: American Diabetes Association criteria (2009); EDCDN: Epidemiology and Diagnostic Criteria of Diabetic Nephropathy; FFXST: Fufang Xueshuantong; UAER: urinary albumin excretion rate; ACR: urine albumin-to-creatinine ratio; mAlb: urine microalbumin; BUN: blood urea nitrogen; HbA1c: hemoglobin A1c; TG: serum triglyceride; LDL-C: low density lipoprotein cholesterol; SBP: systolic blood pressure; CRP: C-reactive protein; qd: quaque die; tid: ter in die.

**Table 2:** GRADE assessment of quality of evidence for outcomes.

Question: should FFXST be used for diabetic kidney disease? Bibliography: Fufang Xueshuanton for diabetic nephropathy. Cochrane database of systematic reviews (year), issue [issue].											
Quality assessment							Summary of findings				
Participants (studies) follow-up	Risk of bias	Inconsistency	Indirectness	Imprecision	Publication bias	Overall quality of evidence	Study event rates (%)		Relative effect (95% CI)	Anticipated absolute effects	
							With control	With FFXS**T**		Risk with control	Risk difference with FFXST (95% CI)
Total effective rate (CRITICAL OUTCOME)											
177 (2 studies)	Serious^1^	No serious inconsistency	No serious indirectness	No serious imprecision	Undetected	⊕⊕⊕⊝ MODERATE^1^ due to risk of bias	56/89 (62.9%)	76/88 (86.4%)	**RR 1.37** (1.15 to 1.64)	Study population	
										**629 per 1000**	**233** more per **1000** (from 94 more to 403 more)
										Moderate	
										**627 per 1000**	**232** more per **1000** (from 94 more to 401 more)

UAER (IMPORTANT OUTCOME; better indicated by lower values)											
383 (5 studies)	Serious^1^	Serious^2^	No serious indirectness	No serious imprecision	Undetected	⊕⊕⊝⊝ **LOW**^1, 2^ due to risk of bias, inconsistency	193	190	**—**		The mean uaer in the intervention groups was **30.98** lower (49.3 to 12.66 lower)

ACR (IMPORTANT OUTCOME; better indicated by lower values)											
638 (6 studies)	Serious^1^	Serious^3^	No serious indirectness	No serious imprecision	Undetected	⊕⊕⊝⊝ LOW^1, 3^ due to risk of bias, inconsistency	318	320	**—**		The mean acr in the intervention groups was **1.33** standard deviations lower (1.9 to 0.76 lower)

mAlb (mg/L) (IMPORTANT OUTCOME; better indicated by lower values)											
548 (6 studies)	Serious^1^	Serious^4^	No serious indirectness	No serious imprecision	Undetected	⊕⊕⊝⊝ **LOW**^1, 4^due to risk of bias, inconsistency	274	274	**—**		The mean malb (mg/l) in the intervention groups was **36.29** lower (54.45 to 18.13 lower)

BUN (mmol/L) (IMPORTANT OUTCOME; better indicated by lower values)											
338 (4 studies)	Serious^1^	Serious^2^	No serious indirectness	Serious^5^	Undetected	⊕⊝⊝⊝ VERY LOW^1, 2, 5^ due to risk of bias, inconsistency, imprecision	167	171	**—**		The mean bun (mmol/l) in the intervention groups was **0.59** lower (1.46 lower to 0.27 higher)

HbA1C (IMPORTANT OUTCOME; better indicated by lower values)											
671 (8 studies)	Serious^1^	No serious inconsistency	No serious indirectness	Serious^4^	Undetected	⊕⊕⊝⊝ LOW^1, 4^ due to risk of bias, imprecision	337	334	**—**		The mean hba1c in the intervention groups was **0.02** lower (0.12 lower to 0.08 higher)

LDL-C (IMPORTANT OUTCOME; better indicated by lower values)											
353 (4 studies)	Serious^1^	No serious inconsistency	No serious indirectness	No serious imprecision	Undetected	⊕⊕⊕⊝ MODERATE^1^ due to risk of bias	175	178	-		The mean ldl-c in the intervention groups was **0.39** lower (0.58 to 0.2 lower)

TG (IMPORTANT OUTCOME; better indicated by lower values)											
811 (9 studies)	Serious^1^	Serious^6^	No serious indirectness	No serious imprecision	Undetected	⊕⊕⊝⊝ **LOW**^1, 6^ due to risk of bias, inconsistency	407	404	**—**		The mean tg in the intervention groups was **0.39** lower (0.63 to 0.15 lower)

SBP (NOT IMPORTANT OUTCOME; better indicated by lower values)											
275 (3 studies)	Serious	Serious^7^	No serious indirectness	Serious^5^	Undetected	⊕⊝⊝⊝ VERY LOW^5, 7^ due to risk of bias, inconsistency, imprecision	138	137	**—**		The mean sbp in the intervention groups was **1.26** lower (3.86 lower to 1.34 higher)

CRP (mg/L) (NOT IMPORTANT OUTCOME; better indicated by lower values)											
148 (2 studies)	Serious^1^	Serious^8^	No serious indirectness	No serious imprecision	Undetected	⊕⊕⊝⊝ LOW^1, 8^ due to risk of bias, inconsistency	74	74	**—**		The mean crp (mg/l) in the intervention groups was **1.92** lower (2.64 to 1.2 lower)

(1) The blind method of the included study was not mentioned. (2) Significant heterogeneity, I2 = 89%. (3) Significant heterogeneity, I2 = 91%. (4) Significant heterogeneity, I2 = 99%. (5) 95% CI crosses the invalid line. (6) Significant heterogeneity, I2 = 84%. (7) Significant heterogeneity, I2 = 58%. (8) Significant heterogeneity, I2 = 68%.

## Data Availability

All data generated or analyzed during the study are available and included in this published article.
